# Exploring the Developmental Overnutrition Hypothesis Using Parental–Offspring Associations and *FTO* as an Instrumental Variable

**DOI:** 10.1371/journal.pmed.0050033

**Published:** 2008-03-11

**Authors:** Debbie A Lawlor, Nicholas J Timpson, Roger M Harbord, Sam Leary, Andy Ness, Mark I McCarthy, Timothy M Frayling, Andrew T Hattersley, George Davey Smith

**Affiliations:** 1 Medical Research Council Centre for Causal Analyses in Translational Epidemiology, University of Bristol, Bristol, United Kingdom; 2 Department of Social Medicine, University of Bristol, Bristol, United Kingdom; 3 Wellcome Trust Centre for Human Genetics, University of Oxford, Oxford, United Kingdom; 4 Department of Oral and Dental Science, University of Bristol, Bristol, United Kingdom; 5 Oxford Centre for Diabetes, Endocrinology and Metabolism, University of Oxford, Oxford, United Kingdom; 6 Genetics of Complex Traits, Institute of Biomedical and Clinical Sciences, Peninsula Medical School, Exeter, United Kingdom; 7 Diabetes Genetics, Institute of Biomedical and Clinical Sciences, Peninsula Medical School, Exeter, United Kingdom; Children's Hospital Boston, United States of America

## Abstract

**Background:**

The developmental overnutrition hypothesis suggests that greater maternal obesity during pregnancy results in increased offspring adiposity in later life. If true, this would result in the obesity epidemic progressing across generations irrespective of environmental or genetic changes. It is therefore important to robustly test this hypothesis.

**Methods and Findings:**

We explored this hypothesis by comparing the associations of maternal and paternal pre-pregnancy body mass index (BMI) with offspring dual energy X-ray absorptiometry (DXA)–determined fat mass measured at 9 to 11 y (4,091 parent–offspring trios) and by using maternal *FTO* genotype, controlling for offspring *FTO* genotype*,* as an instrument for maternal adiposity. Both maternal and paternal BMI were positively associated with offspring fat mass, but the maternal association effect size was larger than that in the paternal association in all models: mean difference in offspring sex- and age-standardised fat mass *z*-score per 1 standard deviation BMI 0.24 (95% confidence interval [CI]: 0.22 to 0.26) for maternal BMI versus 0.13 (95% CI: 0.11, 0.15) for paternal BMI; *p*-value for difference in effect < 0.001. The stronger maternal association was robust to sensitivity analyses assuming levels of non-paternity up to 20%. When maternal *FTO,* controlling for offspring *FTO,* was used as an instrument for the effect of maternal adiposity, the mean difference in offspring fat mass *z*-score per 1 standard deviation maternal BMI was −0.08 (95% CI: −0.56 to 0.41), with no strong statistical evidence that this differed from the observational ordinary least squares analyses (*p* = 0.17).

**Conclusions:**

Neither our parental comparisons nor the use of *FTO* genotype as an instrumental variable, suggest that greater maternal BMI during offspring development has a marked effect on offspring fat mass at age 9–11 y. Developmental overnutrition related to greater maternal BMI is unlikely to have driven the recent obesity epidemic.

## Abstract 2

Using parental-offspring associations and the*FTO* gene as an instrumental variable for maternal adiposity, Debbie Lawlor and colleagues found that greater maternal BMI during offspring development does not appear to have a marked effect on offspring fat mass at age 9-11.

## Editors' Summary

**Background.:**

Since the 1970s, the proportion of children and adults who are overweight or obese (people who have an unhealthy amount of body fat) has increased sharply in many countries. In the US, 1 in 3 adults is now obese; in the mid-1970s it was only 1 in 7. Similarly, the proportion of overweight children has risen from 1 in 20 to 1 in 5. An adult is considered to be overweight if their body mass index (BMI)—their weight in kilograms divided by their height in meters squared—is between 25 and 30, and obese if it is more than 30. For children, the healthy BMI depends on their age and gender. Compared to people with a healthy weight (a BMI between 18.5 and 25), overweight or obese individuals have an increased lifetime risk of developing diabetes and other adverse health conditions, sometimes becoming ill while they are still young. People become unhealthily fat when they consume food and drink that contains more energy than they need for their daily activities. It should, therefore, be possible to avoid becoming obese by having a healthy diet and exercising regularly.

**Why Was This Study Done?:**

Some researchers think that “developmental overnutrition” may have caused the recent increase in waistline measurements. In other words, if a mother is overweight during pregnancy, high sugar and fat levels in her body might permanently affect her growing baby's appetite control and metabolism, and so her offspring might be at risk of becoming obese in later life. If this hypothesis is true, each generation will tend to be fatter than the previous one and it will be very hard to halt the obesity epidemic simply by encouraging people to eat less and exercise more. In this study, the researchers have used two approaches to test the developmental overnutrition hypothesis. First, they have asked whether offspring fat mass is more strongly related to maternal BMI than to paternal BMI; it should be if the hypothesis is true. Second, they have asked whether a genetic indicator of maternal fatness—the “A” variant of the *FTO* gene—is related to offspring fat mass. A statistical association between maternal *FTO* genotype (genetic make-up) and offspring fat mass would support the developmental nutrition hypothesis.

**What Did the Researchers Do and Find?:**

In 1991–1992, the Avon Longitudinal Study of Parents and Children (ALSPAC) enrolled about 14,000 pregnant women and now examines their offspring at regular intervals. The researchers first used statistical methods to look for associations between the self-reported prepregnancy BMI of the parents of about 4,000 children and the children's fat mass at ages 9–11 years measured using a technique called dual energy X-ray absorptiometry. Both maternal and paternal BMI were positively associated with offspring fat mass (that is, fatter parents had fatter children) but the effect of maternal BMI was greater than the effect of paternal BMI. When the researchers examined maternal *FTO* genotypes and offspring fat mass (after allowing for the offspring's *FTO* genotype, which would directly affect their fat mass), there was no statistical evidence to suggest that differences in offspring fat mass were related to the maternal *FTO* genotype.

**What Do These Findings Mean?:**

Although the findings from first approach provide some support for the development overnutrition hypothesis, the effect of maternal BMI on offspring fat mass is too weak to explain the recent obesity epidemic. Developmental overnutrition could, however, be responsible for the much slower increase in obesity that began a century ago. The findings from the second approach provide no support for the developmental overnutrition hypothesis, although these results have wide error margins and need confirming in a larger study. The researchers also note that the effects of developmental overnutrition on offspring fat mass, although weak at age 9–11, might become more important at later ages. Nevertheless, for now, it seems unlikely that developmental overnutrition has been a major driver of the recent obesity epidemic. Interventions that aim to improve people's diet and to increase their physical activity levels could therefore slow or even halt the epidemic.

**Additional Information.:**

Please access these Web sites via the online version of this summary at http://dx.doi.org/10.1371/journal.pmed.0050033.

- See a related *PLoS Medicine* Perspective article
- The MedlinePlus encyclopedia has a page on obesity (in English and Spanish)
- The US Centers for Disease Control and Prevention provides information on all aspects of obesity (in English and Spanish)
- The UK National Health Service's health Web site (NHS Direct) provides information about obesity
- The International Obesity Taskforce provides information about preventing obesity and on childhood obesity
- The UK Foods Standards Agency, the United States Department of Agriculture, and Shaping America's Health all provide useful advice about healthy eating for adults and children
- The ALSPAC Web site provides information about the Avon Longitudinal Study of Parents and Children and its results so far

## Introduction

According to the developmental overnutrition hypothesis, high maternal glucose and high free fatty acid and amino acid plasma concentrations result in permanent changes in appetite control, neuroendocrine functioning, or energy metabolism in the developing fetus and thus lead to greater adiposity and risk of obesity in later life [1–6]. Since greater maternal adiposity is associated with a greater risk of insulin resistance and glucose intolerance, mothers who are more obese at the time of their pregnancy and when breast-feeding will have higher concentrations of glucose and free fatty acids, and the offspring of these mothers would be expected to be programmed to become more obese themselves [2–6]. The consequences of this hypothesis (if true) are important and relate to a pattern of anticipation in the prevalence of obesity and consequent decline in public health: “the obesity epidemic could accelerate through successive generations independent of further genetic or environmental factors” [7].

Two experimental designs for determining the validity and magnitude of the effect of this hypothesis are: (i) to compare the magnitude of the maternal–offspring adiposity association to that of the paternal–offspring adiposity association and (ii) to use genetic variants associated with maternal adiposity as instrumental variables for the causal association of maternal adiposity with offspring adiposity [8].

A similar magnitude of effect of maternal and paternal adiposity on offspring measures of adiposity would suggest that the associations are driven by factors that are just as likely to be passed from father to offspring as they are from mother to offspring (i.e., this would not support the developmental overnutrition hypothesis). Two recent studies have used this approach [9,10]. In an Australian birth cohort, maternal body mass index (BMI) was more strongly associated with offspring BMI than was paternal BMI, and this difference was robust in sensitivity analyses accounting for non-paternity of up to 20% [9]. However, in the Avon Longitudinal Study of Parents and Children (ALSPAC), the associations were the same in mothers and fathers [10]. Both of these studies used BMI as an indicator of offspring adiposity, which is not a good indicator of adiposity in children [11]. We extend this earlier work here by comparing the associations of maternal and paternal BMI with fat mass assessed by dual energy X-ray absorptiometry (DXA) scan in the ALSPAC cohort and by using a genetic variant as an instrumental variable for maternal adiposity.

**Figure 1:**
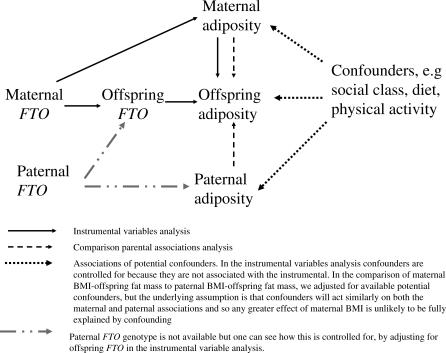
Directed Acyclic Graph Demonstrating the Associations Used to Assess the Developmental Overnutrition Hypothesis in this Study

Common genetic variants that are robustly associated with a modifiable (non-genetic) risk factor can be used to determine the causal effect of this modifiable risk factor on disease risk [12–14]. The theoretical basis for the use of maternal genetic variants (whilst controlling for offspring genotype) as instrumental variables for intergenerational effects has been described previously [12]. It is essential in this approach to adjust for offspring genotype because the association we are interested in is whether greater maternal adiposity during developmental periods is associated with programming of obesity in her offspring (rather than the association of maternal genotype with offspring adiposity per se). Maternal genotype in this approach is simply used as an unconfounded and unbiased instrument for her adiposity. It would not, however, be a valid instrument if we did not adjust for offspring genotype, since an obvious explanation for an association of maternal genotype with offspring genotype would be that the offspring has inherited their mothers' adiposity-related genotype, and that the association is not due to developmental overnutrition (see Figure 1). We are unaware of this approach being used to explore the developmental overnutrition hypothesis to date.

A recent genomewide association study for type 2 diabetes susceptibility identified a variant in the *fat mass and obesity associated* (*FTO*) gene that predisposes to type 2 diabetes via an effect on BMI [15]. We were able to demonstrate an additive effect of the variant on BMI, replicated in 13 unselected independent population studies with 38,759 participants [15]. In ALSPAC offspring, the variant was shown to have a specific association with fat mass [15]. Thus, we are able to use this genotype as an instrumental variable to determine the causal effect of mean differences in maternal BMI on offspring fat mass.

Figure 1 shows a directed acyclic graph (DAG) depicting these two experimental approaches, which were used in this study to assess the validity and likely effect of the developmental overnutrition hypothesis.

The aim of this study was to test the developmental overnutrition hypothesis by (i) determining whether maternal BMI is more strongly related to offspring fat mass than is paternal BMI, and (ii) using maternal *FTO*, whilst controlling for offspring *FTO*, as an instrumental variable for the causal association of maternal BMI on offspring fat mass.

## Methods

### Participants

**Figure 2:**
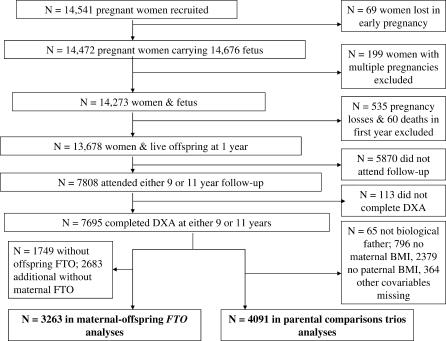
Participants in ALSPAC and in the Analyses Presented in This Paper

The ALSPAC is a longitudinal, population-based birth cohort study that recruited 14,541 pregnant women resident in Avon, United Kingdom, with expected dates of delivery from 1 April 1991 to 31 December 1992. Figure 2 shows a flow of participants through this study from recruitment of pregnant women through to inclusion in the analyses presented in this paper. Additional information on sources of missing data is provided in Text S1 and Table S2. Because intrauterine effects are markedly different for singletons and multiple births, for the purposes of this study we considered only singleton births. There were 13,678 live-born singleton offspring who survived to 1 y of age.

From age 7 y, surviving offspring, with parental consent, were invited to regular follow-up clinics (initially annual and then every second year from age 11 y). For the current study, we used outcome measurements from the two most recent clinics (mean age, 9 and 11 y). In the current study, eligible participants are surviving singletons who attended the 9- or 11-y follow-up examinations in which DXA assessment of total fat and lean mass were undertaken (*N* = 7,221 for the 9-y clinic, *N* = 6,710 for the 11-y clinic, and 7,808 for either clinic). Of these eligible participants, 4,091 (52%) had all relevant data for the parental–offspring trio analyses and 3,263 (42%) had all relevant data for the *FTO* instrumental variable analyses. The study protocol has been described previously [16], and further details are on the ALSPAC website (http://www.alspac.bris.ac.uk). Ethical approval for all aspects of data collection was obtained from the ALSPAC Law and Ethics Committee (IRB 00003312) and the Local Research Ethics Committee (LREC).

### Assessment of Parental BMI and Potential Confounding Factors

Detailed information was obtained from the mother and her partner during pregnancy using self-reported questionnaires. At enrolment, the mother was asked to record her height and pre-pregnancy weight, from which BMI was calculated. She was also asked whether her partner was the father of her unborn child. In the analyses of maternal-paternal-offspring trios, we excluded trios where the mother had reported that her partner was not the biological father of the child and those for whom this information was missing (*N* = 65). For the remainder of the paper, we refer to those included in the analyses as fathers. Age at delivery was derived from date of birth, which was recorded at that time. At the time of recruitment, mothers were also asked to pass a questionnaire to the father of the child; in this questionnaire, the father was asked to record his height and weight, from which BMI (at the start of his partner's pregnancy) was calculated, and also his date of birth, so that his age could be derived.

At enrolment and throughout pregnancy, infancy and childhood questionnaires have been completed by the mother and the father. Maternal parity and the child's sex were obtained from the obstetric records. Mothers were asked about their smoking throughout pregnancy, and these data were used to generate a categorical variable: never smoked prior to or during pregnancy; smoked before or in early pregnancy only (reported not smoking in second and third trimesters); and smoked throughout pregnancy (reported smoking in second and/or third trimester). Breast-feeding was obtained from repeat questions completed by the mother in the first 2 y, and breast-feeding was categorised as: never. <3 mo; 3–6 mo; and >6 mo duration. Based on questionnaire responses, the highest parental occupation was used to allocate the children to family social class groups (classes I [professional/managerial] to V [unskilled manual workers]) using the 1991 British Office of Population and Census Statistics (OPCS) classification. Highest educational qualification for both parents was collapsed into one of five categories from none/CSE (national school exams at age 16) to university degree. Tanner's stages of pubertal development were assigned from mother's responses to mailed line drawings on which they indicated stage of development of their offspring.

### Assessment of Offspring Fat and Lean Mass

Identical protocols were used at both the 9- and 11-y clinics. Current age of the child was recorded in months as they arrived at the assessment clinic. A Lunar prodigy narrow fan beam densitometer was used to perform a whole-body DXA scan in which bone content and lean and fat masses are measured. The scans were visually inspected and realigned where necessary. Once complete, the tester examined the scan to ensure its quality and if necessary repeated the scan. DXA provides valid estimates of fat mass [17]. Height was measured, without shoes, to the nearest 0.1 cm using a Harpenden stadiometer.

### Genotyping

All genotyping of *FTO* (GenBank [http://www.ncbi.nlm.nih.gov/] accession number NM_001080432) was performed by KBioscience (http://www.kbioscience.co.uk). Single nucleotide polymorphisms were genotyped using the KASPar chemistry, which is a competitive allele-specific PCR single nucleotide polymorphism genotyping system using FRET quencher cassette oligos (http://www.kbioscience.co.uk/genotyping/genotyping-chemistry.htm). Blind duplicates, plate-identifying blank wells, and Hardy-Weinberg equilibrium tests were used as quality control tests.

### Statistical Analyses

Absolute fat and lean mass had positively skewed distributions, and their logged (natural logs) values were used in determining correlation coefficients and for calculating age- and sex-standardised *z*-scores. Pearson's pairwise correlation coefficients are presented for the DXA assessed lean and fat mass outcomes at ages 9 and 11 y. Log-transformed absolute fat and lean mass at ages 9 and 11 y were strongly correlated with each other (Table S1). We therefore completed analyses with a combined outcome of 9-y OR 11-y *z*-scores (results using an outcome of either the 9-y measurements only or the 11-y measurements only did not differ substantively from those presented here, and are available from the authors). For all analyses, the outcome was sex and age (in week categories) standard deviation scores (*z*-scores) of fat and lean mass derived from the logged values. In all models, we adjusted for height and height squared (assessed at the same time as DXA scan) so that we had a measure of association with fat and lean mass that was not determined by height.

In order to take account of variations in BMI by sex and age and to account for the much broader span of absolute BMI values for fathers compared to mothers (largely due to the wider age span at birth of fathers compared to mothers), we derived age (in 1-y categories)–standardised *z*-scores s for maternal and paternal BMI. The association between parental BMI and offspring fat or lean mass were assessed using multivariable linear regression. An F-statistic was computed to determine statistical evidence of a difference between maternal and paternal BMI associations on offspring outcomes. To examine the potential role of non-paternity in generating greater associations between maternal BMI and offspring outcomes than paternal BMI and offspring outcomes, given the non-biological relationship between some fathers and their apparent offspring, we conducted a sensitivity analysis modelling the effects of non-paternity rates of between 1% and 20% using the equation given in Text S1.

We used χ^2^ tests to compare prevalences of potential confounding factors by both maternal and offspring *FTO* genotype and by whether the mother was overweight or obese (BMI > 25 kg/m^2^) or not. We used two-stage least squares to fit the instrumental variables models, using the “ivreg2” command in Stata. In these models we assumed an additive genetic model (i.e., maternal BMI increasing linearly with each additional A allele of her *FTO* genotype), since this was demonstrated in our primary paper of the association of this genotype with BMI [15]. Adjustment for offspring genotype in these models was achieved by adding offspring genotype as a continuous score of 0, 1, or 2 risk alleles (again assuming an additive effect). We tested our instrumental variable model in a simulation exercise of a very large dataset. Adjustment of maternal genotype for offspring genotype in the way described above produced the correct known coefficients of the simulated dataset, even when the mode of inheritance was not exactly additive, demonstrating that our model is likely to be unbiased. We compared the instrumental variable estimates to those from ordinary least squares linear regression using the Durbin form of the Durbin-Wu-Hausman statistic. We examined F-statistics from the first-stage regressions to evaluate the strength of the instruments. Values greater than 10 are taken to indicate sufficient strength to ensure the validity of instrumental variable methods [18].

All analyses were conducted using Stata version 9.2.

### Dealing with Missing Data

After excluding trios where the mother's partner might not have been the child's biological father and those with missing data on any covariable included in any model, 4,091 participants (52% of those eligible; i.e., single live birth who attended follow-up) contributed to trio analyses and 3,263 (42%) contributed to the *FTO* genotype analyses (Figure 2). Methods used for examining whether missing data might have biased our findings, and the results of these analyses, are provided in Text S1 and Table S2 . These results suggest that our findings are not importantly biased by missing data.

## Results

Allele frequencies for maternal and offspring *FTO* were identical between those with outcome data and those without outcome data from the original cohort who had genotype data, and there were no departures from Hardy-Weinberg equilibrium (Table S2).

### Parental BMI–Offspring Fat and Lean Mass Comparisons

Table S1 shows correlations between offspring DXA body composition measurements. Total fat mass was strongly correlated with trunkal fat mass, as was total lean mass with trunkal lean mass. There were modest correlations of lean and fat mass. Because of the strong correlations of total fat mass with trunkal fat mass and of total lean mass with trunkal lean mass, all associations with total fat mass were identical to those with trunkal fat mass, and those with total lean mass were identical to those with trunkal lean mass. Results for total fat and lean mass only are presented in all further analyses.

Table 1 presents the associations of parental BMI with offspring fat and lean mass. In all multivariable models, maternal BMI was more strongly associated with offspring fat mass than was paternal BMI with this outcome, with strong statistical evidence for a difference in the parental associations (*p* < 0.001). The association of maternal BMI with offspring lean mass was weaker than that of maternal BMI with offspring fat mass, and the maternal BMI association with offspring lean mass was similar to that of paternal BMI with this outcome. When we repeated the analyses using parental BMI as kg/m^2^ rather than an age- and sex-standardised *z*-scores, the overall meaning of the findings did not differ substantively from those presented in Table 1. For example, in the fully adjusted (equivalent to Model 3) offspring, fat mass increased by 0.06 standard deviation (SD; 95% confidence interval [CI]: 0.05 to 0.07) per 1 kg/m^2^ of maternal BMI and by 0.04 SD (95% CI: 0.03 to 0.05) per 1 kg/m^2^ of paternal BMI (*p* = 0.002 for difference in regression coefficients).

**Table 1:**
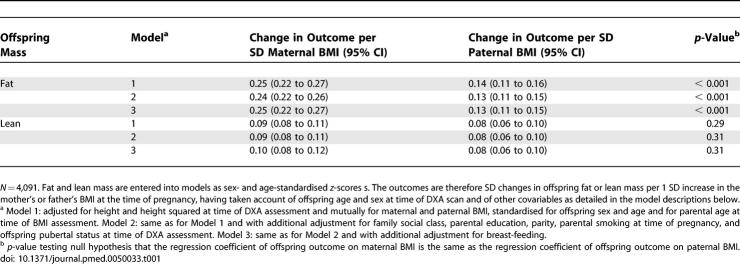
Maternal and Paternal BMI Associations with Offspring DXA-Assessed Fat and Lean Mass at Ages 9 to 11 y

Table S3 shows sensitivity analyses for the maternal–paternal BMI differences in association with offspring fat mass assuming different levels of non-paternity. These suggest that the stronger maternal BMI–offspring fat mass association, compared to paternal BMI–offspring fat mass association, is robust to assumed levels of non-paternity up to 20%, though the difference attenuates. In all analyses the maternal–paternal difference is small, amounting to an absolute greater maternal BMI effect on offspring fat mass of 0.11 SD of offspring fat mass per 1 SD maternal BMI compared to 1 SD paternal BMI. This difference reduces to 0.08 at assumed levels of non-paternity of 10% and 0.05 at assumed levels of 20%.

### Associations of *FTO* Genotype with Potential Confounders and with Adiposity

Neither maternal nor offspring *FTO* genotypes were associated with any of the potential confounding factors that might confound the association of maternal BMI with offspring fat or lean mass (Table 2). In contrast, mothers who were overweight or obese at the start of pregnancy were more likely to be from manual social classes, to have lower educational attainment, and to have a partner (the father of the child) with lower educational attainment and to be parity 3 or greater, compared to women with healthy BMI (Table 2).

**Table 2:**
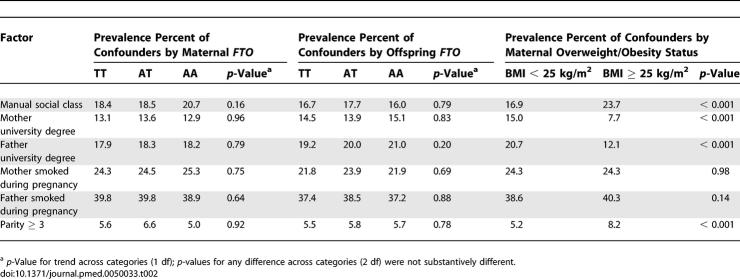
Association of Maternal and Offspring *FTO* Genotype and Maternal Overweight/Obesity with Potential Confounding Factors

Our findings for the associations of maternal and offspring *FTO* with BMI and fat mass in those included in our main analyses here were essentially the same as those presented in our earlier paper in which maximum samples were used [15]. Maternal *FTO* was associated with maternal BMI: for each additional A allele, there was a linear increase in BMI of 0.09 SD (95% CI: 0.04 to 0.14) in our sample. Offspring's *FTO* was associated with their total fat mass: 0.13 SD (95% CI: 0.09 to 0.18) per allele, and only weakly with their lean mass: 0.03 SD (95% CI: 0.00 to 0.06) per allele. The association of offspring *FTO* with their fat mass was the same for those children whose mothers had normal weight at the start of pregnancy (BMI < 25 kg/m^2^) compared to those who were overweight or obese (BMI ≥ 25 kg/m^2^): 0.13 SD (95% CI: 0.08 to 0.18) versus 0.11 SD (95% CI: 0.04 to 0.18) per allele; *p*-value for interaction with maternal overweight/obese status = 0.87.

### Analyses Using *FTO* as an Instrumental Variable for Greater Maternal Adiposity

**Figure 3:**
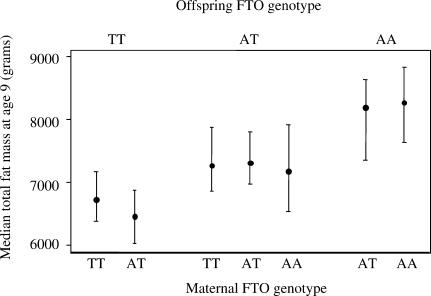
Median Total Fat Mass by Offspring and Maternal *FTO* Genotype

Table 3 shows the analyses in which *FTO* is used as an instrumental variable for maternal adiposity throughout her lifespan. In order to highlight the importance of controlling for offspring *FTO* to fulfill the conditions for maternal *FTO* to be an instrumental variable (as shown in Figure 1), we present two sets of results—those that do not control for offspring *FTO* and those with control for offspring *FTO*. In analyses that do not control for offspring genotype, the instrumental variable analyses are consistent with the ordinary least squares analyses and suggest that greater maternal BMI is causally related to greater offspring fat mass. With adjustment for offspring *FTO*, there is no specific effect of maternal BMI on offspring fat mass. The first stage F-statistic for the instrumental variables analyses without adjustment for offspring *FTO* was 12.9, and 10.1 with adjustment for offspring *FTO.* The findings from our instrumental variables analyses are further illustrated in Figure 3, which shows median offspring total fat mass at age 9 y by maternal and offspring genotype (results for fat mass at age 11 y were the same). Fat mass increases with each additional offspring A allele within all categories of maternal genotype, but maternal genotype does not affect offspring fat mass within strata of offspring genotype.

**Table 3:**
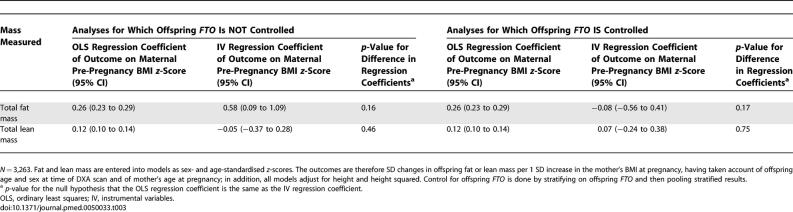
Association of Maternal BMI with Offspring Fat and Lean Mass Assessed at Ages 9 to 11 y Using *FTO* Genotype as an Instrumental Variable for Maternal BMI

## Discussion

In this study, maternal pre-pregnancy BMI was positively associated with offspring fat mass assessed at age 9 or 11 y, with this association being stronger than the association of maternal BMI with offspring lean mass. The association of maternal BMI with offspring fat mass was stronger than the association of paternal BMI with offspring fat mass, even in sensitivity analyses that take account of plausible levels of non-paternity. These findings suggest that at least a part of the maternal BMI–offspring fat mass association is related to factors that are specific to the mother. A plausible maternal specific effect is overnutrition during key developmental periods (intrauterine and during breast-feeding), and hence the findings of this part of the study are supportive of the developmental overnutrition hypothesis.

The maternal–paternal difference is small. Assuming no non-paternity in this sample, and that the parental differences are completely explained by development overnutrition, our findings would suggest that the offspring of mothers who have a pregnancy BMI greater by 1 SD than other mothers will have on average 0.11 SD greater fat mass at ages 9 to 11 y as a result of developmental overnutrition. If we accept that there might be non-paternity of up to 10%, then this reduces to 0.08 SD. Effects of this magnitude would account for only a very small proportion of the obesity epidemic. For example, among United Kingdom children aged 11–16 y, BMI increased by 0.47 SD in males and 0.53 SD in females between 1987 and 1997 [19], five times the difference we note here between maternal–offspring and paternal–offspring associations. Effects of this magnitude across many generations could result in a slow and steady increase in population levels of obesity, something that might have been occurring from the early part of the 20th century [20]. However, the recent obesity epidemic has taken the shape of a major increase in mean BMI and obesity prevalence over a period of 10–15 y (as described above). This time period would be the equivalent of one generation, and therefore the weak specific maternal effect is unlikely to have made a major impact on the obesity epidemic.

Analyses that used maternal *FTO* genotype as an instrumental variable for maternal BMI and adjusted for offspring genotype did not show an association of maternal BMI with offspring fat mass. Maternal *FTO* (with control for offspring *FTO*) is an unconfounded proxy for maternal adiposity across all of her lifespan, including when she was pregnant and breast-feeding her offspring [12]. Thus, these findings do not provide strong evidence in favour of the developmental overnutrition hypothesis. Despite the point estimate from these analyses being very close to the null value, the confidence intervals were wide, and statistically these findings were consistent with those of the maternal–paternal comparison. Taken together, the findings from our parental comparisons analyses and *FTO* instrumental variable analyses would be consistent with a relatively weak specific effect of greater maternal BMI during pregnancy on offspring adiposity at ages 9–11 y.

The instrumental variable analysis uses the proportion of the variation in maternal BMI that is explained by *FTO* to provide an estimate of causal effect that is not biased by confounding. However, the advantage of this approach being less biased than a conventional multivariable regression analysis comes at the cost of reduced precision (i.e., wide confidence intervals). The imprecision in our instrumental variable analyses highlights the importance of large sample sizes in order to obtain precise estimates of effects in studies that use genotype as an instrumental variable for modifiable non-genetic exposures [21]. To our knowledge, no other study of larger magnitude than ours has both maternal and offspring genotype and objectively measured fat mass in offspring, and we feel combining both the approach of parental–offspring association comparisons and the use of a genetic variant known to be associated with adiposity as an instrumental variable for maternal adiposity is a strength of this paper.

Our genetic results are unlikely to be biased by missing data. This is because substantial empirical evidence demonstrates that selection bias (i.e., associations in those who volunteer to be in a study and/or who remain during follow-up being different from those who do not enter or remain in a study, and hence different from the population that one is making inference about) is very unlikely to affect genetic associations [22,23] (Table S2), which shows that genotype distribution does not vary by whether offspring have follow-up DXA scan outcomes or not. For example, in the recently published genome-wide association study from the Wellcome Trust Case Control Consortium, all allele frequencies from a control group made up of British blood donors (a group who are known to vary considerably from the general population in terms of non-genetic factors such as socioeconomic position and lifestyle) were the same as those from a control group taken from the British 1958 birth cohort, a general population sample [24]. Sensitivity analyses that explored the potential bias due to missing data in either sets of analyses suggest that this is minimal (see Text S1).

The developmental overnutrition hypothesis refers to the effect of delivery to the developing fetus (or infant during the early postnatal period) of higher concentrations of glucose and free fatty acids. Levels of these nutrients are likely to be greater in women with greater adiposity, and therefore there is a concern that women with greater amounts of adipose tissue will program their offspring to be more obese [8]. However, our findings cannot be used to draw inferences about the specific role of maternal levels of glucose and free fatty acids during the intrauterine period and whilst she is breast-feeding on later offspring adiposity and metabolic health. Maternal gestational diabetes is associated with later offspring obesity and diabetes risk [2,25–31], and there is some evidence of a graded association across maternal distributions of fasting and postload glucose during pregnancy with offspring obesity risk at ages 5–7 y [32]. Thus, the question of whether there is a graded causal association between greater delivery of glucose and free fatty acids during development on later obesity requires further exploration using robust study designs.

The public health importance of the developmental overnutrition hypothesis is that, if it is true, the obesity epidemic could accelerate across generations and continue to do so for some time, even with effective obesity prevention programmes. This is because the female offspring of mothers with greater adiposity during their pregnancy would be programmed to greater adiposity and would enter their own pregnancies with greater adiposity and continue the cycle. In this study, we have examined associations between two generations (mothers and their offspring) only and shown that specific maternal effects appear weak and unlikely to have made a major contribution to the recent obesity epidemic (see above). If a specific maternal effect on offspring adiposity of ∼0.1 SD per maternal SD adiposity were replicated across many generations, this effect could make a contribution to a slow and steady increase in obesity over many decades. Of importance, it is unclear at what time in the life course developmental overnutrition will have its maximum effect. In other examples of developmental origins epidemiology, there is evidence that associations amplify with increasing age. For example, a recent large study demonstrated amplification of the association between birth weight and blood pressure with increasing age [33]. We have only been able to measure offspring outcomes at mean age 9–11 y here, but have plans to continue to measure fat and lean mass as the offspring enter adulthood. If any specific maternal affect increases with the increasing age of her offspring, such that it was particularly strong when the female offspring were themselves in their reproductive years, then developmental overnutrition could have made an important contribution to the obesity epidemic. This suggestion requires further study in older offspring. Finally, our findings cannot exclude an association between greater maternal weight gain during pregnancy and increased adiposity in later life in her offspring.

A stronger effect of greater maternal adiposity than paternal adiposity on offspring fat mass could reflect the greater role of mothers in childhood nutrition and feeding habits, rather than a developmental origins effect. Whilst this is a possibility, surprisingly few studies have examined differences in maternal–offspring and paternal–offspring behaviours. In a study using data form the Norwegian National Health Survey, both maternal and paternal dietary fat intake were strongly associated with offspring dietary fat intake, but the magnitude of association was the same for mothers and fathers [34]. By contrast, the mother's level of exercise had a much weaker effect on offspring's exercise levels (whatever the sex of the offspring) than did the father's exercise levels in that study [34]. Similarly, in a recent study of Australian families, the fathers' exercise levels had a stronger effect on both their sons' and daughters' exercise levels and objective measures of cardio-respiratory fitness than did the mothers' exercise levels [35]. Thus, there does not appear to be strong evidence in the literature that mothers have a stronger effect on offspring diet and physical activity (behaviours that would affect offspring fat mass) than do fathers.

At this stage, the exact mechanisms by which *FTO* results in increased BMI are not known. Consequently, we cannot discount it having an effect via dietary and physical activity behaviours. If the effect of *FTO* on adiposity was via increased dietary intake or lower physical activity levels, and maternal behaviours such as diet and activity were more strongly influential on such behaviours in offspring, then we would anticipate an association of maternal *FTO*, adjusted for offspring *FTO*, with offspring fat mass (one mediated via maternal behaviour rather than developmental overnutrition), but we do not see such an association in this study.

One could conceive that even with a gene that predisposes to greater adiposity, social pressures to be thinner might mean that any effect is compensated for by lifestyle modification, which would then prevent its use as an instrumental variable for adiposity [21]. This possibility is easily tested by demonstrating a robust association between the genetic variant (or other instrumental variable) and the intermediate of interest [21]. In the case of *FTO,* its robust association with BMI and fat mass, including in this study, has been demonstrated [15]. Furthermore, in separate work we have demonstrated that *FTO* is associated with insulin resistance, hyperglycaemia, dyslipidaemia, and hyptertension to the extent that one would predict from observational studies of the association of BMI with these outcomes and of randomised trials of weight change interventions with these outcomes [36]. If *FTO* were not a valid instrument of adiposity, one would not expect such accurate prediction of the causal effect of adiposity on outcomes that are widely accepted as being causally related to greater adiposity [36].

Since the mechanisms by which *FTO* is related to greater adiposity are currently unknown, it is possible that pleiotropy could explain our null instrumental variables finding. To do so, maternal *FTO* would have to affect other pathways in such a way that these pathways counterbalance the hypothesised positive effect of maternal BMI on offspring fat mass. Maternal imprinting could also theoretically bias *FTO* as a valid instrumental variable for maternal adiposity. However, since none of the known imprinted human genes are on Chromosome 16 [37], where *FTO* is located, this is very unlikely. Population stratification is unlikely in this sample because the participants, on the basis of self-reported place of birth and parental and grandparental places of birth, are largely ancestrally homogeneous (of European origin).

In conclusion, our findings suggest that any specific effect of maternal greater BMI during offspring development on offspring fat mass at ages 9–11 y is at most weak and unlikely to be a major driver of the recent obesity epidemic.

## Supporting Information

Table S1 Correlations of Log Transformations of DXA-Assessed Fat and Lean Mass at Ages 9 and 11 y (39 KB DOC)

Table S2 Genotype and Allele Frequencies of Offspring and Mothers by Whether the Offspring has DXA-Assessed Fat and Lean Mass at Ages 9 and 11 y (37 KB DOC)

Table S3 Sensitivity Analyses of Comparison of Maternal BMI–Offspring Fat Mass with Paternal BMI-Offspring Fat and Lean Mass Taking Account of Different Levels of Possible Non-Paternity (59 KB PDF)

Text S1 Additional Methodological Details and Sensitivity Analyses (32 KB DOC)

## Abbreviations

ALSPAC: Avon Longitudinal Study of Parents and Children
BMI: body mass index
CI: confidence interval
DXA: dual energy X-ray absorptiometry
SD: standard deviation
