# Supplementary material for: Exploring the Developmental Overnutrition Hypothesis Using Parental–Offspring Associations and FTO as an Instrumental Variable
Source: PLoS Med. 2008 Mar 11;5(3):e33. doi: 10.1371/journal.pmed.0050033 (PMC2265763; doi:10.1371/journal.pmed.0050033)
Supplement: Supplementary file 1 [file pmed.0050033.sd001.doc]

**Text S1: Additional methodological details and sensitivity**

**analyses.**

**Methods use for sensitivity analyses taking account of different potential levels of non-paternity**

As stated in the main paper, we excluded from the analyses family groups where it was stated that the partner of the mother was not the child’s biological father. However, it has been suggested that unknown rates of non-paternity may affect up to 20% of births in general populations. In analyses where the effect of maternal exposure on an outcome is compared to paternal exposure on an outcome, false differences (stronger associations in mothers) may occur because of possible unknown non-paternity. The extent to which this might affect results can be examined statistically by assuming different levels of non-paternity in a particular sample. To assess this we undertook a series of sensitivity analyses using a modified version of the equation initially suggested by Clemons [1], and that has been used in two previous papers that have compared effects of maternal and paternal BMI on offspring BMI to test the developmental overnutrition hypothesis [2,3]. The equation is given below:

Λ= $\left( \begin{matrix} \sigma\text{ff} & a\sigma\text{fm} \\ a\sigma\text{fm} & \sigma\text{mm} \end{matrix} \right)$ ${}^{{\begin{aligned} -1 \end{aligned}}}$ $\left( \begin{matrix} (1-p)\sigma\text{ff} & (1-p)\sigma\text{fm} \\ \sigma\text{fm} & \sigma\text{mm} \end{matrix} \right)$

where	$\sigma\text{ff}$	= variance of reported father’s body mass index

$\sigma\text{mm}$	= variance of mother’s body mass index

$\sigma\text{fm}$	= covariance of reported father’s and mother’s body mass index

*p*	= probability that the reported father is not the biological father

*a*	is used to indicate the possible covariances between the

mother’s and biological father’s body mass index; we assumed it to be equal to the covariance between the mother’s and the reported father’s BMI, and used *a* = 1.

The observed regression coefficients were multiplied by Λ^-1^ to obtain the modified coefficients given in Table S3. That is the results for differences between maternal-offspring associations and paternal-offspring associations, assuming different possible levels of unknown non-paternity.

**Assessment of possible effects of missing data on results**

Figure 2 in the main paper provides a flow of participants in the study, from recruitment of pregnant women through to those included in the analyses presented in this paper. In the current study eligible participants are those who attended the 9 and 11 year follow-up examinations where DXA assessment of total fat and lean mass were undertaken (N = 7,221 for the 9 year clinic and 6,710 for the 11 year clinic; 7808 who attended either). Of these 6,867 (95%) at the 9 year clinic and 6565 (98%) at the 11 year clinic completed the DXA scan (N = 7695 had a scan at one or other of the clinic). For the parental BMI-offspring fat and lean mass associations we excluded participants whose mothers had indicated that her partner was not the child’s biological father or who had not completed this question (N=65), those who had missing data on either mothers (N=796) or fathers BMI (N = 2379) or any of the potential confounding factors. In total 3,724 participants contributed to analyses with DXA outcomes at age 9; 3,658 to analyses with outcomes at age 11 and 4,091 to outcomes at 9 or 11 (representing 52-60% of those eligible for outcomes at different ages).

In total (i.e. of all singleton offspring who were alive at 1 year) 9067 live singleton offspring had DNA sent for *FTO* genotyping and off these 8446 (93%) were successfully genotyped. 8480 mothers of live singleton offspring had DNA sent for genotyping and of these 8055 (95%) were successfully genotyped. Of these mothers and offspring with genotype data there were 5685 dyads with *FTO* data for both mother and offspring. For the *FTO* genotype analyses we excluded participants with missing genotype data on either mother or offspring and missing DXA scan fat or lean mass. In total 2,964 participants contributed to analyses with DXA outcomes at age 9; 2,840 to analyses with outcomes at age 11 and 3,263 to outcomes at 9 or 11 (representing 41-45% of those eligible for outcomes at different ages). Most missing data was more common for maternal *FTO* genotype, with 72-74% of offspring having genotype for each of the associations with outcomes at different ages but just 59% having maternal *FTO* and 41-45% having both.

For both sets of analyses those included compared to those excluded because of missing data did not differ with respect to maternal or paternal BMI or offspring lean mass (all p-values > 0.2). For both sets of analyses those included had slightly lower fat mass than those included in analyses: mean difference in fat mass comparing those included in the *FTO* gene analyses to those excluded because of missing data -0.05 standard deviation (95%CI: -0.09, 0.00), with a similar magnitude of difference for the parental BMI-offspring outcomes analyses.

In addition to the main analyses presented in the paper, analyses were also conducted separately for maximum samples in any individual model. For example, we analysed maternal BMI-offspring outcome associations in those with complete data on maternal BMI and offspring DXA outcomes irrespective of whether they had data on paternal BMI and all potential confounders. The results from these maximum samples were essentially identical to those presented here. Similarly, we examined the association of offspring *FTO* genotype with offspring fat and lean mass at all ages without restricting these analyses to those who also had valid data for maternal genotype. When we compared these results to the results obtained amongst the subgroup who also had maternal genotype they were identical, but had somewhat larger standard errors due to the smaller sample size.

Finally, allele frequencies for maternal and offspring *FTO* were identical between those with DXA outcome data and those without DXA outcome data from the original cohort, but who had genotype data, and there were no departures from Hardy-Weinberg equilibrium (Table S1). The similarity in genotype frequency between those with and without DXA is to be expected since genotype should not affect selection, and is consistent with a number of studies that have empirically shown that selection bias should not be a problem for genetic association studies [4-6]. For example, in the recently published genome-wide association study from the Wellcome Trust Case Control Consortium, all allele frequencies from a control group made up of British blood donors (a group who are known to vary considerably from the general population in terms of non-genetic factors such as socioeconomic position and lifestyle) were the same as those from a control group taken from the British 1958 birth cohort, a general population sample [6].

# References

(1)	Clemons T (2000) A look at the inheritance of height using regression toward the mean. *Hum Biol* 72:447-454.

(2)	Lawlor DA, Davey Smith G, O'Callaghan M, Alati R, Abdullah M, et al. (2007) Epidemiological evidence for the fetal overnutrition hypothesis: findings from the Mater-University study of pregnancy and its outcomes. *Am J Epidemiol* 165:418-424

(3)	Davey Smith G, Leary S, Steer C, Ness AR (2007) The intergenerational acceleration hypothesis for increasing obesity levels: evidence from parent-offspring associations in the Avon Longitudinal Study of Parents and Children (ALSPAC). *Arch Dse Childhood* 92:876-880

(4)	Bhatti P, Sigurdson AJ, Wang SS, Chen J, Rothman N, et al. (2005) Genetic variation and willingness to participate in epidemiologic research: data from three studies. *Cancer Epidemiol Biomarkers Prev* 14:2449-2453.

(5)	Davey Smith G, Lawlor DA, Harbord R, Timpson N, Day I, et al. (2007) Clustered environments and randomized genes: a fundamental distinction between conventional and genetic epidemiology. *PloS Medicine* In press.

(6)	The Wellcome Trust Case Control Consortium (2007) Genome-wide association study of 14,000 cases of seven common diseases and 3,000 shared controls. Nature 447:661-678
