# Supplementary material for: Exploring the Developmental Overnutrition Hypothesis Using Parental–Offspring Associations and FTO as an Instrumental Variable
Source: PLoS Med. 2008 Mar 11;5(3):e33. doi: 10.1371/journal.pmed.0050033 (PMC2265763; doi:10.1371/journal.pmed.0050033)
Supplement: Supplementary file 2 [file pmed.0050033.st001.doc]

Table S1: Correlations of log transformations of DXA assessed fat and lean mass at ages 9 and 11.

|  | Total fat mass at 9 | Total lean mass at 9 | Trunkal fat mass at 9 | Trunkal lean mass at 9 | Total fat mass at 11 | Total lean mass at 11 | Trunkal fat mass at 11 | Trunkal lean mass at 11 |
| --- | --- | --- | --- | --- | --- | --- | --- | --- |
| Total fat mass at 9 | 1 |  |  |  |  |  |  |  |
| Total lean mass at 9 | 0.39 | 1 |  |  |  |  |  |  |
| Trunkal fat mass at 9 | 0.99 | 0.37 | 1 |  |  |  |  |  |
| Trunkal lean mass at 9 | 0.37 | 0.98 | 0.36 | 1 |  |  |  |  |
| Total fat mass at 11 | 0.93 | 0.36 | 0.91 | 0.34 | 1 |  |  |  |
| Total lean mass at 11 | 0.45 | 0.86 | 0.44 | 0.83 | 0.42 | 1 |  |  |
| Trunkal fat mass at 11 | 0.91 | 0.33 | 0.92 | 0.31 | 0.99 | 0.40 | 1 |  |
| Trunkal lean mass at 11 | 0.42 | 0.83 | 0.41 | 0.86 | 0.39 | 0.98 | 0.37 | 1 |

All p-values for these correlations < 0.001
