# Supplementary material for: Exploring the Developmental Overnutrition Hypothesis Using Parental–Offspring Associations and FTO as an Instrumental Variable
Source: PLoS Med. 2008 Mar 11;5(3):e33. doi: 10.1371/journal.pmed.0050033 (PMC2265763; doi:10.1371/journal.pmed.0050033)
Supplement: Supplementary file 3 [file pmed.0050033.st002.doc]

**Table S2: Genotype and allele frequencies of offspring and mothers by whether the offspring has DXA assessed fat and lean mass at ages 9 and 11**

|  | Offspring genotype and allele N (proportion) in those with phenotype | Offspring genotype and allele N (proportion) in those without phenotype | Maternal genotype and allele N (proportion) in those with phenotype | Maternal genotype and allele N (proportion) in those without phenotype |
| --- | --- | --- | --- | --- |
| **Phenotype = DXA outcomes at age 9** | | | | |
| TT | 1980 (0.367) | 1130 (0.368) | 1565 (0.364) | 1338 (0.356) |
| AT | 2558 (0.474) | 1469 (0.478) | 2067 (0.481) | 1821 (0.485) |
| AA | 855 (0.159) | 474 (0.154) | 665 (0.155) | 599 (0.159) |
| T | 6518 (0.604) | 3729 (0.607) | 5197 (0.605) | 4497 (0.598) |
| A | 4268 (0.396) | 2417 (0.393) | 3397 (0.395) | 3019 (0.402) |
| HWE p-value | 0.55 | 0.94 | 0.70 | 0.63 |
| **Phenotype = DXA outcomes at age 11** | | | | |
| TT | 1855 (0.364) | 1255 (0.372) | 1493 (0.365) | 1410 (0.356) |
| AT | 2439 (0.477) | 1588 (0.471) | 1974 (0.482) | 1914 (0.483) |
| AA | 802 (0.157) | 527 (0.156) | 628 (0.153) | 636 (0.161) |
| T | 6149 (0.603) | 3729 (0.607) | 4960 (0.606) | 4734 (0.598) |
| A | 4043 (0.397) | 2417 (0.393) | 3230 (0.394) | 3186 (0.402) |
| HWE p-value | 0.99 | 0.94 | 0.58 | 0.77 |
