# Supplementary material for: Exploring the Developmental Overnutrition Hypothesis Using Parental–Offspring Associations and FTO as an Instrumental Variable
Source: PLoS Med. 2008 Mar 11;5(3):e33. doi: 10.1371/journal.pmed.0050033 (PMC2265763; doi:10.1371/journal.pmed.0050033)
Supplement: Supplementary file 4 [file pmed.0050033.st003.doc]

**Table S3: Sensitivity analyses of comparison of maternal BMI-offspring fat mass with paternal BMI-offspring fat and lean mass taking**

**account of different levels of possible non-paternity.**

For all associations the coefficients are adjusted as in model 3 of table 2 and the outcomes are based on DXA measurements at 9 OR 11 year follow-up. N=4091

| **Assumed prevalence of non-paternity (%)** | **Fully adjusted regression coefficients of offspring fat mass on:** | | **P difference in parental effect** |
| --- | --- | --- | --- |
|  | Maternal BMI | Paternal BMI |  |
| 0 | 0.236 | 0.127 | < 0.001 |
| 1 | 0.236 | 0.129 | < 0.001 |
| 2 | 0.236 | 0.132 | < 0.001 |
| 3 | 0.235 | 0.134 | < 0.001 |
| 4 | 0.235 | 0.136 | < 0.001 |
| 5 | 0.235 | 0.139 | < 0.001 |
| 6 | 0.234 | 0.141 | < 0.001 |
| 7 | 0.234 | 0.143 | < 0.001 |
| 8 | 0.234 | 0.146 | < 0.001 |
| 9 | 0.233 | 0.148 | < 0.001 |
| 10 | 0.233 | 0.151 | < 0.001 |
| 11 | 0.233 | 0.154 | < 0.001 |
| 12 | 0.232 | 0.156 | < 0.001 |
| 13 | 0.232 | 0.159 | < 0.001 |
| 14 | 0.232 | 0.162 | 0.001 |
| 15 | 0.231 | 0.165 | 0.001 |
| 16 | 0.231 | 0.168 | 0.002 |
| 17 | 0.230 | 0.171 | 0.004 |
| 18 | 0.230 | 0.174 | 0.006 |
| 19 | 0.230 | 0.178 | 0.010 |
| 20 | 0.229 | 0.181 | 0.017 |
